# Pediatric Obstructive Sleep Apnea Syndrome: Time to Wake Up

**DOI:** 10.5005/jp-journals-10005-1134

**Published:** 2012-02-24

**Authors:** Veena Arali, Srinivas Namineni, Ch Sampath

**Affiliations:** Senior Lecturer, Department of Pedodontics and Preventive Dentistry Sri Sai College of Dental Sciences, Vikarabad, Andhra Pradesh, India e-mail: arali.veena@gmail.com; Professor and Head, Department of Pedodontics and Preventive Dentistry, Sri Sai College of Dental Sciences, Vikarabad, Andhra Pradesh, India; Professor, Department of Pedodontics and Preventive Dentistry Sri Sai College of Dental Sciences, Vikarabad, Andhra Pradesh, India

## Abstract

Pediatric patients with well-controlled OSA present few difficulties for routine dental treatment. However, patients with untreated or undiagnosed OSA can present the dental practitioner with multiple issues and challenges. Dental professionals have a unique doctor-patient relationship that affords them a role in recognizing sleep disorders by exploring the history of patients who are sleepy.

**Aim:** This paper is aimed at providing comprehensive review of pediatric obstructive sleep apnea.

**How to cite this article:** Arali V, Namineni S, Sampath Ch, Pediatric Obstructive Sleep Apnea Syndrome: Time to Wake Up. Int J Clin Pediatr Dent 2012;5(1):54-60.

## INTRODUCTION

The stupid-lazy child who frequently suffers from headaches at school, breathes through his mouth instead of his nose, snores and is restless at night, and wakes up with a dry mouth in the morning, is well worthy of the solicitous attention of the school medical officer. This nineteenth- century reference was published in an article entitled ‘On some causes of backwardness and stupidity in children’ and describes the unfortunate sequelae resulting from lack of recognition and treatment for the common disease we have now know as the pediatric obstructive sleep apnea syndrome (OSAS).^1^

OSAS was initially described in the medical literature in 1966 as a breathing disorder during sleep affecting an obese adult male. At this time, the pediatric medical community had not yet recognized the diagnosis of OSAS; however, several pediatric cases of hypertrophied tonsils and adenoids causing congestive heart failure and cor pulmonale were reported.^2^ Although the sleep breathing disorders in children were recognized early, it was not until 1976 that the first case reports of obstructive sleep apnea in children were published.^3^ Since then, OSAS in children has been the focus of much attention and research and is now widely accepted as a significant cause of morbidity in childhood.^1^

OSAS in childhood, as defined by the American Thoracic Society, is a disorder of breathing during sleep characterized by prolonged partial upper airway obstruction and/or intermittent complete obstruction, obstructive apnea, that disrupts normal ventilation during sleep and normal sleep patterns.^4^

## EPIDEMIOLOGY

No definitive population-based study has evaluated the presence of OSAS in children. The percentage of individuals younger than 18 years who have been reported with regular heavy snoring oscillated between 8 and 12%. Initial studies estimated OSAS prevalence to be between 1 and 3%. More recently, many specialists have estimated OSAS prevalence to be between 5 and 6%.^5^

To understand the pathophysiology of OSAS in children it is important to understand the physiology of breathing and sleep.

There is an immediate increase in upper airway resistance with the sleep onset, with an initial ‘overshoot’ in this resistance that decreases very quickly. Still, this resistance during established sleep is mildly higher than during wakefulness.^6^ There is also a slight decrease in tidal volume with sleep. This decrease will be more pronounced with the occurrence of rapid eye movement (REM) sleep. These mild decreases will be compensated by a slight increase in breathing frequency to keep minute ventilation normal. Breathing frequency decreases during the first 2 years of life but stays the same thereafter; it has been calculated to range from a maximum of 16 to 18 breaths/ minute in non-REM sleep and 17 to 19 breaths/minute during REM sleep.^7^

The obesity epidemic, evident in the United States and industrialized countries, has complicated the investigation of obstructive sleep apnea (OSA) and related syndromes. Fat distribution varies according to genetic, sex and hormonal patterns and the inherent relationship among these three factors. It is common for fat to deposit in the abdominal region. Such abdominal obesity will lead to chest-bellows impairment, as seen in restrictive thoracic disorders. Although it may not lead to upper airway obstruction, abdominal obesity may worsen the poor gas exchange that may already exist because of OSAS. Sleep will always worsen the gas exchange in these subjects.

When they are in the supine position and when they achieve REM sleep. During REM sleep, the associated atonia eliminates contractions of the accessory respiratory muscles and the abdominal muscles, which engage in active expiration.^7^ Also, REM sleep is associated with further flattening of the diaphragm’s position.^2^ These physiological changes worsen gas exchange in subjects with abdominal obesity and may even lead to REM sleep-related hypoventilation with some degree of carbon dioxide (CO_2_) retention. Upper airway impairment, *per se*, is not directly related to this CO_2_ retention. It has, however, been hypothesized that abnormal gas exchange during sleep may impair the coordination of time-related contractions of both upper airway dilator muscles and inspiratory muscles.^5^

## PATHOPHYSIOLOGY

### Role of the Tonsils and Adenoids

Adenotonsillar hypertrophy clearly plays a role in the pathogenesis of childhood OSAS. Other causes of childhood OSAS includes obesity, craniofacial disease and neuro- muscular disease; however, these are less common.^8^

Vast majority of children with OSAS has large tonsils and adenoids. It was found that site of upper airway closure was at the level of the tonsils and adenoids, whereas in normal children, it was at the level of the soft palate.^9^ Most obvious is the fact that patients with OSAS do not obstruct during wakefulness, when the tone of the upper airway muscles is increased. However, many studies have failed to show a correlation between upper airway or adenotonsillar size and OSAS.^10,11^

### Role of Upper Airway Neuromotor Tone

Although the overall ventilatory drive appears to be normal in children with OSAS, it is possible that central augmentation of upper airway neuromotor function is abnormal. The upper airway muscles are accessory muscles of respiration, such as are activated by stimuli such as hypoxemia, hypercapnia^12^ and upper airway subatmospheric pressure. The tendency of the upper airway to collapse is inversely related to the level of activity of the upper airway dilator muscles.^13-15^ Therefore, increased upper airway neuromotor tone may be one way that patients can compensate for a narrow upper airway, which has been shown in adults during wakefulness. This compensatory mechanism was lost during sleep.^16^

Unlike adults children have a less collapsible upper airway than normal adults. The upper airway neuromotor activation in children is regulated by various factors including central ventilator drive, chemoreceptor afferents, upper airway pressure and flow receptors, pulmonary mechanoreceptors and sleep state. The central nervous system is known to play a crucial role in maintaining upper airway patency.^8^ Experiments have demonstrated the potential of the pediatric upper airway to modulate airflow in response to such a stimuli as subatmospheric pressure and CO_2_. Furthermore, the studies suggest that children not only have increased basal upper airway tone during sleep, but that the tone can be increased even further in response to a stimulus. Thus, pharyngeal muscle activity appears to play a prominent role in preserving upper airway patency in children during sleep, in order to compensate for an anatomically smaller upper airway.^8^

### Role of Arousal

The arousal response to obstructive apnea differs markedly between children and adults. Arousals frequently do not occur in children. In infants, less than 20% of obstructive apneas was associated with arousal.^17^ This lack of cortical arousal in response to airway obstruction probably accounts for the lack of sleep fragmentation and resultant daytime somnolence in pediatric patients. It may also explain why children can go on to have extended, uninterrupted periods of obstructive hypoventilation.

In general, children have a higher arousal threshold than adults; the younger the child, the higher the arousal threshold.^18^

### Role of Other Structural Factors

Structural factors other than adenotonsillar hypertrophy may play a role in the pathogenesis of childhood OSAS. Doubtless children with craniofacial anomalies are at risk for OSAS. However, it is unclear to what degree minor anatomical differences can contribute to sleep-disordered breathing.^8^

### Role of Genetic Factors

Genetic factors play a role in the pathophysiology of OSAS, as demonstrated by studies of family cohorts. It is unclear whether this is due to the modulating influence of genetic factors on the ventilatory drive,^19,20^ anatomic features^21,22^ or both.

## CLINICAL SYMPTOMS

The clinical symptoms are known to vary with age. Recognition of the problem is often only noted in older children, who are able to articulate complaints. The parental complaints of children seen at sleep clinics overtime include (Table 1).^8^

## CLINICAL EVALUATION AND DIAGNOSIS OF SDB

The scepticism of sleep-disordered breathing (SDB) indicates the need not only for a general pediatric evaluation but also for a thorough evaluation of the upper airway anatomy. Clinically, it involves a comprehensive examination of its successive segments.

**Table 1:** **Table 1: **Characteristic features of sleep apnea in different age groups

*Infants, 3-12 months*		*Toddlers, 1-3 years*		*Preschool-aged children*		*School-aged children*	
Disturbed nocturnal sleep with repetitive crying Poorly established day/night cycle Noisy breathing or snoring Nocturnal sweating Poor suck Absence of normal growth pattern or failure to thrive Observation of apneic events Report of apparent lifethreatening event Presence of repetitrive earaches or URI		Noisy breathing or snoring Agitated sleep or disrupted nocturnal sleep Crying spells or sleep terrors Grouchy and/or aggressive daytime behavior Daytime fatigue Nocturnal sweating Mouth breathing Poor eating or failure to thrive Repetitive URI Witnessed apneic episodes		Regular, heavy snoring Mouth breathing Drooling during sleep Agitated sleep Nocturnal awakenings Confusional arousals Sleepwalking Sleep terrors Nocturnal sweating Abnormal sleeping positions Persistence of bed-wetting Abnormal daytime behavior Aggressiveness Hyperactivity Inattention Daytime fatigue Hard to wake up in the morning Morning headache Increased need for napping compared with peers Poor eating Growth problems Frequent URI		Regular, heavy snoring Agitated sleep Abnormal sleeping positions Insomnia Delayed sleep phase syndrome Confusional arousal Sleepwalking, sleep talking Persistence of bed-wetting Nocturnal sweating Hard to wake up in the morning Mouth breathing, drooling Morning headache Daytime fatigue Daytime sleepiness with regular napping Abnormal daytime behaviors Pattern of attention-deficit/hyperactivity disorder Aggressiveness Abnormal shyness with drawn and depressive presentation Learning difficulties Abnormal growth patterns Delayed puberty Repetitive URI Dental problems appreciated by dentist Crossbite Malocclusion (class II or III) Small jaw

1. The nose, one should look for asymmetry of the nares, a large septal base, collapse of the nasal valves during inspiration, a deviated septum or enlargement of the inferior nasal turbinates.
2. The oropharynx should be examined for the position of the uvula in relation to the tongue. The scale developed by Mallampati et al^23^ scale may help to evaluate this position.
3. The size of the tonsils should be compared with the size of the airway.^24^
4. The presence of a high and narrow hard palate, overlapping incisors, a crossbite and an important (2 mm) overjet (the horizontal distance between the upper and lower teeth) are indicative of a small jaw and/or abnormal maxilla-mandibular development.^8^

## OBJECTIVE CONFIRMATION OF SDB

Testing during sleep is the only way to confirm the presence of SDB. Controversy exists concerning the need for and type of test to be performed. Some of the measures used for this testing include questionnaires and scales, home monitoring and PSG.^25-28^

Although questionnaires may be helpful in directing the attention of parents to the diurnal and nocturnal symptoms of SDB, the sensitivity and specificity of the questionnaires are not sufficient for affirming the presence of SDB.^29-31^

Home monitoring with or without videotaping has also been used. Ambulatory monitoring with recording of cardiac and respiratory variables has been suggested as the first diagnostic step in testing for SDB. These devices can detect the presence of drops in oxygen saturation (SaO_2_), apneas and hypopneas; affirm the diagnosis of SDB; and lead to treatment.^32-34^

Polysomnography is the only test that may exclude the diagnosis of SDB. It must always include monitoring of sleep/wake states through electroencephalography (EEG), electro-oculography, chin and leg electromyography, electrocardiography, body position and appropriate monitoring of breathing.^8^

### Polysomnographic Differences between Children and Adults with OSAS^35^ (Table 2)

The American Thoracic Society has defined their criteria for an abnormal PSG in children as follows:

- Apnea index (AI) 1/hour
- Apnea-hypopnea index 5/hour
- Peak end-tidal carbon dioxide 53 mm Hg or
- An end-tidal carbon dioxide tension 50 mm Hg for 10% of the sleep period and
- A minimum hemoglobin oxygen saturation 92%.

**Table 2:** **Table 2: **Diagnostic criteria of OSAS in children^35^

*Frequent signs*		*Infrequent signs*	
• Nocturnal snoring		• Daytime sleepiness	
• Mouth breathing		• Decreased appetite	
• Restless sleep with or without arousals		• Failure to thrive	
		• Frequent vomiting	
• Respiratory pauses		• Swallowing dysfunction	
• Respiratory infections		• Behavioral problems	
• Chronic rhinorrhea		• Otitis media	
• Nocturnal sweating		• Enuresis	

### Orofacial Implications

**Fig. 1:**
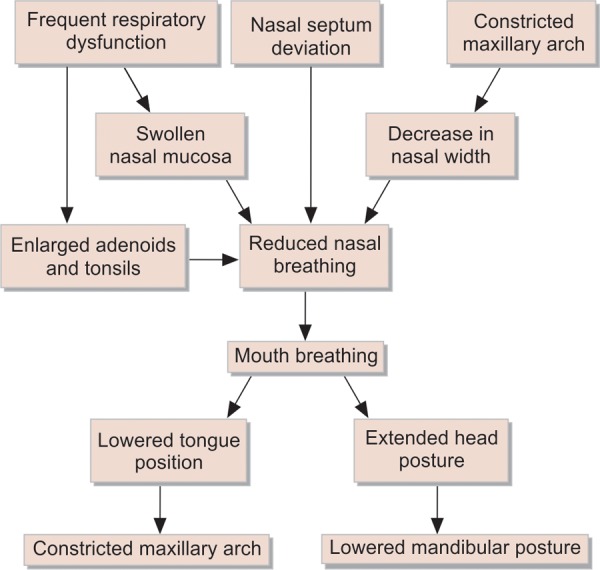
Influences on orofacial growth

It is also clear that the well-described but extremely complex interaction between nasal breathing and facial growth is important, even if it is rarely investigated (Fig. 1).^5^

The most common orofacial characteristics encountered include a retrognathic mandible, narrow palate, large neck circumference, long soft palate (which leads to dentists’ being unable to visualize the entire length of the uvula when the patient’s mouth is open wide), tonsillar hypertrophy, nasal septal deviation and relative macroglossia.^36^

The following features are found in OSA patients on a cephalogram:^37^

- An increased incidence of maxillary retrusion (ANB < 0)
- An increased incidence of mandibular retrusion (ANB > 0)
- An increased incidence of maxillary and mandibular retrusion (SNA and SNB)
- The hyoid was more inferiorly and anteriorly placed
- A thicker soft palate
- A larger tongue; a longer pharyngeal length.

### Treatment of Obstructive Sleep Apnea in Children^1^

- Adenotonsillectomy
- Medical therapies
  – Nasopharyngeal airway
  – Insufflations of pharynx during sleep
  – Continuous positive airway pressure via nasal mask
- Pharmacological
  – Topical nasal steroids
  – Antibiotics
  – Nasal decongestants
  – Weight loss
- Other surgical therapies
  – Craniofacial surgical procedures
  – Mandibular/maxillary plastic surgical procedures
  – Stenting procedures for nasal stenosis
  – Cleft palate revision procedures
  – Uvulopalatopharyngoplasty
- Tracheostomy.

There is an overall consensus that children with SDB should undergo evaluation by an otolaryngologist for surgical treatment.

### Adenotonsillectomy^5^

Treatment for short-term outcomes indicates that adenotonsillectomy with or without radiofrequency treatment of nasal inferior turbinates is the first approach to consider. A controversial issue is how early to perform adenotonsillectomy. Most will agree that adenotonsillectomy is often performed by 24 months of age. However, OSA has been noted as early as 3 weeks of age, and cases of heavy snoring and clinical symptoms in children aged 6 to 24 months are actually common. Adenotonsillectomy has been performed as early as 6 months of age.

### Orthodontic Treatment

Rapid maxillary distraction (RMD) is an orthodontic technique that is based on the bone formation process. A distractor anchored to two molars on both sides applies daily pressure, pushing apart both halves of the maxilla; bone then grows from the borders of the cartilage. This technique pushes the soft tissues laterally, decreases the height of the soft palate, and enlarges the nasal orifices. Rapid maxillary distraction may be associated with distraction of the mandible, but because no mid cartilage is present, there is very limited widening.

Slow maxillary distraction is based on similar principles and optimizes the degree of widening at the different growth periods that occur in prepubertal children. Rapid and slow maxillary distractions are performed between 5 and 11 years of age. Distraction results in widening of the palate and the nose; thus, these procedure remedies nasal occlusion related to a deviated septum, for which little can be done before 14 to 16 years of age.^5^

### Oral Appliances

Oral appliances have been recommended for the treatment of OSA. Based on a large body of high-level evidence-based studies,^38^ the American Academy of Sleep Medicine recently has published a newly revised practice parameter paper that lends substantial importance and credibility to the use of oral appliances (OAs) in the management of obstructive sleep apnea (OSA)^.39^

A diversity of appliances have been developed to increase the oropharyngeal airway space. They have included devices with extensions to the soft palate or to the base of the tongue, tongue repositioning devices or more commonly, MRA.^40^

Tongue repositioning device works on the principle that the tongue is secured anteriorly by negative pressure from a soft plastic bulb between the lips and teeth but the device is bulky and causes considerable mandibular opening.^41^

The principle behind MRA is that the mandible is held forward during sleep. In so doing, by holding the tongue and pharyngeal muscles forward, the posterior airway space is increased. Mandibular repositioning appliances have the advantage of being simple, reversible and cost-effective. The effects of MRA on the oropharyngeal airway have been well demonstrated by cephalometric radiography and fluoroscopy.^42^

A range of minor side effects with OA’s have been reported including temporomandibular disorders (TMD) symptoms, excessive salivation, dry mouth, bruxism, tooth movement and gingival irritation. Generally, all these side effects are mild in comparison to the complications of obstructive sleep apnea and can be reversed by adjustment or discontinuing the device.^37^

## SURGICAL TREATMENT

Surgeries, such as nasal septoplasty and other maxillofacial surgeries, are indicated in some rare cases but not usually seen in the pediatric population. Orthognathic surgery is normally postponed until 10 to 13 years of age.^5^

Two surgical techniques used in patients with SDB are mandibular distraction osteogenesis and maxillomandibular advancement.

Mandibular distraction osteogenesis is very similar to RMD, but it is applied to the mandible when a maxillary and mandibular widening is needed and when the slow mandibular orthodontic distraction cannot achieve the needed result.^43^

Maxillomandibular advancement is a very successful procedure. Nonetheless, it is major surgery that should be performed after there has been apt orthodontic treatment. It may be performed at anytime during childhood, but it is often postponed until 11 to 12 years of age.^43^

### Continuous Positive Airway Pressure (CPAP)

Mechanically bypassing the obstruction with continuous positive airway pressure (CPAP) and bilevel positive airway pressure (BiPAP) has been used successfully in children, although a difficulty in finding appropriately fitting equipment may contribute to problems with adherence. It should be noted that this method is only palliative in nature and does not cure the underlying cause of the obstruction.^5^

### Sequelae of OSAS in Children^1^

- Cardiopulmonary:
  –Right ventricular hypertrophy
  –Left ventricular hypertrophy
  –Pulmonary hypertension
  –Systemic hypertension
  –Cor pumonale
  –Polycythemia
- Neurodevelopmental:
  –Developmental delay
  –Hypersomnolence
  –Poor school performance
  –Leaning problems
  –Hyperactivity
  –Mood and behavior problems.

## CONCLUSION

OSAS in children is a rather new diagnostic entity and several issues regarding diagnosis, treatment and sequelae are not yet well studied. The timing of initial testing for children known to be at high-risk of OSAS has not been identified. Behavior and learning difficulties associated with OSAS and the effects of early diagnosis and treatment on these specific problems have not yet been well studied. The pathophysiology of childhood OSAS remains poorly understood. Yet, it is thought to be caused by a combination of anatomic and neuromotor factors, i.e. by the superimposition of structural abnormalities upon an inherently more collapsible upper airway. The anatomic disharmonies can be identified at an early age in order to deliver effective treatment to prevent the late outcomes of OSA. The behavior of the child in the dental office may be an alarming sign for identification of OSA. Timely diagnosis is dependent on maintaining an index of suspicion for high- risk children and on establishing a set of routine screening questions regarding sleep habits that can be easily incorporated into routine pediatric dentistry practice.
